# Pressure-controlled fibrinolytic irrigation for membranous and recurrent chronic subdural hematomas

**DOI:** 10.1016/j.bas.2026.105953

**Published:** 2026-02-02

**Authors:** Marco Bissolo, Roberto Doria-Medina, Mazin Omer, Theresa Bettina Loidl, Zeynep Mercan, Mukesch Johannes Shah, Theo Demerath, Eva Rohr, Klaus-Jürgen Buttler, Jürgen Beck, Roland Roelz

**Affiliations:** a Department of Neurosurgery, Medical Center - University of Freiburg, Faculty of Medicine, University of Freiburg, Freiburg, Germany; b Department of Neuroradiology, Medical Center - University of Freiburg, Faculty of Medicine, University of Freiburg, Freiburg, Germany

**Keywords:** Chronic subdural hematoma, TDC-FIT, Treatment recurrence, Fibrinolysis, Irrigation, Recurrence, Minimally invasive

## Abstract

**Introduction:**

Chronic subdural hematoma (cSDH) is a common neurosurgical condition with substantial recurrence after conventional evacuation. In June 2023, we introduced twist-drill craniostomy with pressure-controlled fibrinolytic irrigation therapy (TDC-FIT), developed for patients at elevated recurrence risk.

**Research question:**

This study evaluates the safety and effectiveness of TDC-FIT in reducing cSDH recurrence compared with standard approaches.

**Methods:**

We performed a retrospective comparative analysis of all consecutive cSDH procedures from January 2021 to December 2024, including twist-drill craniostomy (TDC), open craniotomy (OC), and TDC-FIT. The primary endpoint was reoperation within six months for symptomatic recurrence or inadequate evacuation—defined as residual hematoma ≥10 mm, midline shift, or persistent symptoms. Secondary endpoints included perioperative complications and outcome.

**Results:**

A total of 779 procedures were performed in 491 patients: 698 TDC (89.6%), 40 OC (5.1%), 34 TDC-FIT (4.4%), and 7 others. Overall recurrence per procedure was 30.0%. Hematoma-related membranes strongly predicted recurrence (OR 3.4; p < 0.0001). Recurrence after TDC-FIT was 8.8%, significantly lower than TDC (30.1%) and OC (25.0%) (p = 0.024). In membranous hematomas, recurrence reached 45.0% after TDC, 24.3% after OC, and 9.7% after TDC-FIT (p = 0.001). Predictive modeling matched observed recurrence for TDC but markedly overestimated recurrence for TDC-FIT and OC. Adverse events were lowest after TDC (7.4%) and TDC-FIT (8.8%) and highest after OC (20.0%). Over time, practice shifted toward minimally invasive techniques with increasing adoption of TDC-FIT.

**Conclusions:**

TDC-FIT demonstrated a favorable safety profile and substantially reduced recurrence compared with conventional techniques, supporting its integration as an effective minimally invasive alternative.

## Highlights

• Chronic subdural hematoma remains burdened by high recurrence despite conventional surgical treatment.
• Patients with membranous hematomas are particularly vulnerable to recurrence and poor radiographic resolution.
• Introducing TDC-FIT provides a targeted minimally invasive strategy for these high-risk cases.
• TDC-FIT shows lower recurrence than twist-drill craniostomy or open craniotomy, even in membranous hematomas.
• Its favorable safety profile and effectiveness are driving a progressive shift toward TDC-FIT in our clinical practice.

## 1 Introduction

Chronic subdural hematoma (cSDH) is a common neurosurgical condition in elderly patients, often complicated by high postoperative recurrence despite standard evacuation procedures (Feghali et al., 2020). Recurrence frequently necessitates repeated interventions, prolonging hospitalization, increasing morbidity, and raising healthcare costs, while correlating with poorer recovery and long-term disability (Rauhala et al., 2020).

Current therapeutic approaches emphasize adjunctive measures to reduce recurrence risk. Middle meningeal artery (MMA) embolization has shown promise in recent randomized trials, yet it remains resource-intensive, requiring specialized endovascular expertise, dedicated infrastructure, and procedural availability. (Liu et al., 2024; Davies et al., 2024; Fiorella et al., 2025; Shotar et al., 2025). Still, no standard exists for recurrence prophylaxis, and surgical reintervention remains the mainstay.

Twist-drill craniostomy (TDC) offers a minimally invasive approach suitable for bedside performance under local anesthesia, particularly advantageous for elderly patients with significant comorbidities (Reinges et al., 1998). However, TDC historically shows elevated recurrence rates compared to more invasive approaches like burr-hole craniostomy (BHC) and open craniotomy (OC), which demonstrate superior efficacy but carry increased procedural risks, require general anesthesia and extended recovery times (Yagnik et al., 2021; Huang et al., 2022).

To balance efficacy and safety, our institution introduced twist-drill craniostomy with pressure-controlled fibrinolytic irrigation therapy (TDC-FIT) in June 2023 (Bissolo et al., 2025a, 2025b; Doria-Medina et al., 2025). This technique combines the minimally invasive approach of TDC with pressure-regulated urokinase irrigation to facilitate the targeted degradation of neomembranous components contributing to hematoma persistence. Pressure-controlled fibrinolytic irrigation could disrupt this process by enhancing clearance of organized collections and promoting collapse of the hematoma cavity, potentially reducing the risk of reaccumulation. Furthermore, the pressure-controlled system may mitigate the high risk of rebleeding in compartmentalized, membranous cSDH by preventing abrupt intracranial pressure shifts that could otherwise lead to membrane detachment or cortical collapse.

Early results from 16 patients suggested lower reoperation rates and effective hematoma resolution with safety comparable to TDC and passive drainage (Doria-Medina et al., 2025).

This study reviews all cSDH interventions at our center over four years, comparing TDC-FIT with standard approaches. We evaluate recurrence, reoperation, morbidity, adverse events (AE), and functional outcomes to determine its potential as a safe and effective alternative.

## 2 Material and methods

### 2.1 Study design and oversight

We conducted a retrospective single-center cohort study of cSDH surgeries at a tertiary neurosurgical center between January 1, 2021, and December 31, 2024. The study was approved by the institutional review board (approval no. 24–1201-S1) and complied with the Declaration of Helsinki. All patient data were anonymized before analysis.

### 2.2 Outcomes

The primary endpoint was reoperation within six months of index surgery for symptomatic recurrence or inadequate evacuation, defined as residual hematoma ≥10 mm (or exceeding calvarial thickness), midline shift, or persistent symptoms. Secondary endpoints were perioperative complications, functional outcome (modified Rankin Scale, mRS) at final follow-up, and perioperative mortality. Recurrence rates were reported per procedure rather than per patient to enable accurate comparison across interventions.

### 2.3 Data collection

Clinical data were extracted retrospectively from electronic health records, operative reports, and radiological archives. Variables included patient age and sex, hematoma laterality, presence of membranes on imaging, and preoperative antithrombotic therapy (vitamin K antagonists, direct oral anticoagulants (DOACs), antiplatelet agents, or dual antithrombotic therapy). Surgical modality and subsequent postoperative interventions were recorded. Treatment related AE were classified per Common Terminology Criteria for Adverse Events (CTCAE). Data on adjunctive treatments such as MMA embolization and management of spinal cerebrospinal fluid (CSF) leaks were also collected.

### 2.4 Surgical procedures and IRRAflow® and LiquoGuard® 7 systems

All cSDH surgeries were included, with modality selected based on presentation, imaging, comorbidities, and recurrence. Procedures were performed by board-certified neurosurgeons according to institutional standards. OC involved craniotomy, hematoma evacuation, membrane resection if indicated, and subdural drainage, followed by ICU monitoring. TDC was performed at bedside under local anesthesia with a burr hole and single-lumen drainage, usually without ICU care. After craniostomy, the single-lumen drain was left in place for about 48 h in most cases. The drain remained until the output decreased, at which point it was flushed with 2 mL of saline and withdrawn by 2–3 cm. A follow-up CT scan was performed on postoperative day 2 to ensure adequate hematoma evacuation, after which the drain was removed. TDC-FIT, introduced in June 2023 for recurrent or high-risk cSDH, combined standard TDC with continuous, pressure-controlled urokinase irrigation via a dual-lumen IRRAflow® catheter or two single-lumen catheters connected to LiquoGuard® 7. This allowed regulated infusion and drainage to promote fibrinolysis while preventing pressure shifts. Initially managed in the ICU, since 2024 stable patients have been treated on the general ward.

The IRRAflow**®** system operates in repeating cycles of irrigation, pressure monitoring, and drainage. Integrated safety features continuously track ICP to prevent excessive irrigation or overdrainage. If ICP rises above the preset upper threshold, irrigation is automatically suspended and the system switches to drainage-only mode for approximately 2 min while alerting the user. Conversely, if ICP falls below the lower threshold, both irrigation and drainage are paused, and an alert is issued.

Similarly, the LiquoGuard**®** 7 system includes high and low ICP alarms to maintain drainage within safe limits. This system allows continuous irrigation alongside passive drainage. Physicians can set the operational pressure range, which typically spans from −1 mmHg to 19 mmHg. Infusion rates can also be adjusted: during daytime, rates generally range between 50 and 100 mL/h, while overnight infusion is reduced to approximately 10 mL/h to minimize pressure-related complications in a standard ward setting.

Nursing staff were trained to continuously monitor both IRRAflow**®** and LiquoGuard**® 7** systems and to notify medical personnel if any issues arose. Preparation of the urokinase infusion was performed by the medical team. Continuous physician presence or intermediate care monitoring was not required solely due to the use of these systems, unless the patient's overall clinical condition dictated otherwise.

The LiquoGuard**®** 7 system was used off-label in patients at high risk of recurrence or with prior recurrences, following a detailed informed discussion and offered as an individual therapeutic attempt in accordance with institutional regulations.

### 2.5 Statistical analysis

Statistical analyses were conducted using Microsoft Excel (v16.88), Wizard Pro (v1.9.42), and GraphPad Prism (v10.2.3). Categorical variables were compared using chi-square or Fisher's exact tests as appropriate. Continuous variables were analyzed via the Mann–Whitney *U* test. All tests were two-sided, with a significance threshold of p < 0.05. Results are reported as odds ratios (OR) with 95% confidence intervals (CI) when applicable.

To assess the independent effect of surgical technique on recurrence risk, we developed a multivariable logistic regression model using patient characteristics known to influence chronic subdural hematoma recurrence. The model was constructed using data from all patients treated with TDC to establish baseline recurrence risk under usual care. Patients treated with TDC-FIT and open craniotomy were excluded from model development because these treatments were applied selectively and had limited covariate overlap with TDC, increasing the risk of confounding by indication. The fitted model was then applied to the other treatment groups to generate expected 6-month recurrence risk for risk-adjusted comparisons.

Four covariates were selected a priori based on clinical relevance and prior literature: patient age (analyzed as a continuous variable), sex, antithrombotic medication status, and presence of membranes on preoperative imaging. Antithrombotic therapy was categorized into five mutually exclusive groups: no therapy, antiplatelet agents alone, vitamin K antagonists, direct oral anticoagulants, and dual antithrombotic therapy. Membrane presence was defined as visible internal septations, layering, or compartmentalization within the hematoma cavity on computed tomography, suggestive of organized or loculated hematoma structure.

The primary outcome was hematoma recurrence within 6 months after surgery, defined as symptomatic reaccumulation requiring reoperation, residual hematoma thickness of 10 mm or greater (or exceeding calvarial thickness), radiographic evidence of midline shift, or persistence of neurologic symptoms attributable to the hematoma.

Multivariable logistic regression was performed using maximum likelihood estimation. Continuous variables were standardized, and categorical variables were appropriately encoded using indicator variables. Model discrimination was assessed using the area under the receiver operating characteristic curve (AUC). Calibration was evaluated by comparing predicted probabilities with observed recurrence rates across risk strata.

Individual recurrence probabilities were calculated for each patient using the fitted model coefficients. These predicted risks enabled risk stratification and provided a baseline expectation for recurrence rates based solely on patient characteristics. The model was subsequently applied to the TDC-FIT cohort to assess whether observed outcomes differed from those predicted by traditional risk factors alone.

## 3 Results

### 3.1 Population characteristics

Over four years, 491 patients underwent 779 cSDH procedures (mean 1.59 per patient); 284 (57.8%) had a single intervention, 207 (42.2%) multiple, and 60 (12.2%) three or more. Most patients were male (325; 66.2%), with slightly more left-sided hematomas (427/779; 54.8%). Membrane formation was observed in 393 procedures (50.4%) on preoperative CT imaging.

The most common leading symptom among all procedures was paresis (35.2%), followed by personality changes (15.1%), headache (14.8%), aphasia (13.2%), decreased level of consciousness (10.1%), other or incidental findings (7.8%), and seizures (3.5%). Symptoms were unspecified in 0.3% of cases. When analyzed at the patient level (n = 491), the distribution remained similar: paresis (32.4%) was the leading symptom, followed by headache (15.5%), aphasia (14.1%), personality change (13.6%), decreased consciousness (10.2%), incidental findings (9.8%), and seizures (4.1%). Unspecified symptoms were recorded in 0.4% of patients.

Most procedures (474; 60.8%) were in patients not on anticoagulation; among anticoagulated patients, 149 (19.1%) received antiplatelet therapy, 103 (13.2%) DOACs, 42 (5.4%) vitamin K antagonists, and 11 (1.4%) dual therapy.

Spinal CSF loss was associated with cSDH in 29 procedures (28 patients; 3.7%), 27 due to spontaneous intracranial hypotension (SIH) requiring repair. One case occurred postoperatively after intraoperative dural tear during the resection of a giant calcified thoracic disc herniation. One patient required two SIH-related surgeries. Baseline characteristics are summarized in Table 1.

**Table 1:** Table 1 summarizes the baseline characteristics of the study cohort and compares key preoperative variables across the three main surgical techniques.

Baseline Characteristics				
**Total Procedures**	779			
**Total Patients**	491			
**Mean Procedures per Patient**	1.59			
**Single Surgery (%)**	284 (57.8%)			
**Multiple Surgeries (%)**	207 (42.2%)			
**Three or More Surgeries (%)**	60 (12.2%)			
**Male Patients (%)**	325 (66.2%)			
**Left Hemisphere (%)**	427 (54.8%)			
**Membrane Formation (%)**	393 (50.4%)			
**TDC Procedures (%)**	698 (89.6%)			
**OC Procedures (%)**	40 (5.1%)			
**TDC-FIT Procedures (%)**	34 (4.4%)			
**BHC Procedures (%)**	1 (0.1%)			
**MMA Embolization (%)**	24 (3.1%)			

	**TDC (n = 697)**	**TDC-FIT (n = 34)**	**OC (n = 40)**	**p-value**

**Age (years), mean ± SD**	77.5 ± 11.8	76.2 ± 14.1	77.2 ± 12.8	0.82
**Male gender, n (%)**	472 (67.7%)	23 (67.6%)	30 (75.0%)	0.62
**Preoperative mRS, median (IQR)**	3 (2–4)	2 (1–3)	3 (2–4)	0.18
**Left laterality, n (%)**	381 (54.7%)	17 (50.0%)	23 (57.5%)	0.81
**Antithrombotic therapy, n (%)**	289 (41.5%)	6 (17.6%)	7 (17.5%)	<0.001
**Membrane present, n (%)**	320 (45.9%)	31 (91.2%)	37 (92.5%)	<0.001

Abbreviations**:** BHC, burr-hole craniotomy; MMA, meningeal medial artery; OC, open craniotomy; TDC, twist-drill craniostomy; TDC-FIT, twist-drill craniostomy with pressure-controlled fibrinolytic irrigation therapy.

### 3.2 Surgical techniques

TDC under local anesthesia was the predominant technique, used in 698 of 779 procedures (89.6%), including 151 of 225 recurrent hematomas (67.1%). OC was performed in 40 cases (5.1%), exclusively after less invasive failures, mainly for organized or multiloculated recurrent hematomas (36/40; 90%), and BHC was used in only one case (0.1%). TDC-FIT, introduced in June 2023, was performed in 34 cases (4.4%), mostly for recurrent hematomas (28/34; 82.4%) and 6 for high-risk primary hematomas. Six OCs addressed acute SDH as a TDC complication. MMA embolization was performed in 24 cases (3.2%) for recurrence, with no subsequent recurrences. Membranes were present in 280/698 TDC procedures (40.1%), 36/40 OC (90%), and 31/34 TDC-FIT (91.2%). Procedure characteristics—including age, laterality, and sex—were comparable across the surgical techniques; however, the presence of membranes and anticoagulation status varied between groups (Table 1).

### 3.3 Total recurrence

Of 779 procedures, 225 (28.9%) were recurrent; recurrence data were missing in 29 cases (4 declined follow-up, 25 unknown), leaving 750 surgeries for analysis with a recurrence rate of 30.0%.

Membrane formation on preoperative imaging was a significant risk factor for recurrence (OR 3.4; 95% CI 2.4–4.8; p < 0.0001). Age, sex, and standard anticoagulation (antiplatelet, DOAC, vitamin K antagonists) did not demonstrate any significant association with recurrence. However, dual antithrombotic therapy was associated with the highest recurrence rate (80.0%), though the sample size was small (n = 11).

Among the patients who experienced recurrence, 12.9% were diagnosed with spinal CSF leaks, including spontaneous spinal leaks, i.e. SIH in the majority (n = 27), and one postoperative CSF leakage. Recognition of a patient as SIH-positive was associated with a substantial reduction in recurrence risk, with an absolute risk reduction of 23.7%. likely due to a change in management and the initiation of targeted treatment for the underlying leak (Overstijns et al., 2025).

### 3.4 Recurrence rate among surgical techniques

TDC was the predominant surgical technique in recurrent SDH, being used in 151 of 225 recurrence procedures (67.1%). Among these 151 recurrences, 125 represented first recurrences after TDC, 22 were second recurrences (21 after TDC and 1 after TDC-FIT), and 4 were third recurrences (3 after OC and 1 after TDC-FIT). OC was performed in 40 cases (5.1%), exclusively after failure of less invasive strategies including 16 first recurrences after TDC, 15 s recurrences after TDC, and 8 third recurrences (3 after TDC and 5 after OC); one patient underwent OC for a fourth recurrence following 2 TDC procedures and 1 OC. Overall, 28 recurrent cases were managed with TDC-FIT, comprising 21 first recurrences after TDC, 5 s recurrences after TDC, and 2 third recurrences (1 after TDC and 1 after OC). MMA embolization was performed in 24 cases (3.2%) for recurrent SDH (1 after TDC-FIT, 1 after OC, and the remainder after TDC), with no further recurrences observed.

Overall recurrence rates varied significantly among surgical groups (p = 0.024). TDC demonstrated the highest recurrence rate at 30.1% (210 of 698 patients), followed by OC at 25.0% (10 of 40 patients), and TDC-FIT at 8.8% (3 of 34 patients) (Fig. 1A).

**Fig. 1A:**
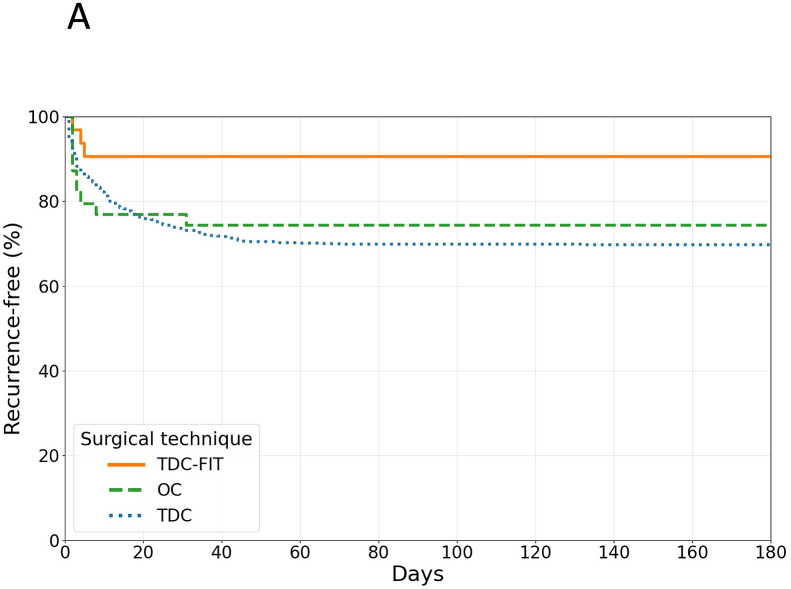
Kaplan-Meier curves showing recurrence-free survival by surgical technique over 180 days. TDC-FIT (orange line) demonstrates superior recurrence-free survival compared to OC (green line) and TDC (blue line). The curves suggest that subdural irrigation therapy may reduce recurrence risk in cSDH treatment (p = 0.024). Abbreviations: OC, open craniotomy; TDC, twist-drill craniostomy; TDC-FIT, twist-drill craniostomy with pressure-controlled fibrinolytic irrigation therapy.

Compared to TDC, TDC-FIT had a lower recurrence risk (OR 0.22; 95% CI 0.06–0.70; p = 0.006), despite higher predicted baseline risk. (See: **Predicted risk model and actual recurrence rate**).

In patients with membranous hematoma, recurrence was 45.0% for TDC (144/320), 24.3% for OC (9/37), and 9.7% for TDC-FIT (3/31; p < 0.001). TDC-FIT was superior to TDC in this subgroup (OR 0.13; 95% CI 0.04–0.40; p < 0.001; absolute RR 35.3%, relative RR 78.5%, NNT 2.8). Compared to OC, TDC-FIT showed a non-significant trend toward lower recurrence (OR 0.33; 95% CI 0.08–1.32; p = 0.20; relative RR 60.2%) (Fig. 1B).

**Fig. 1B:**
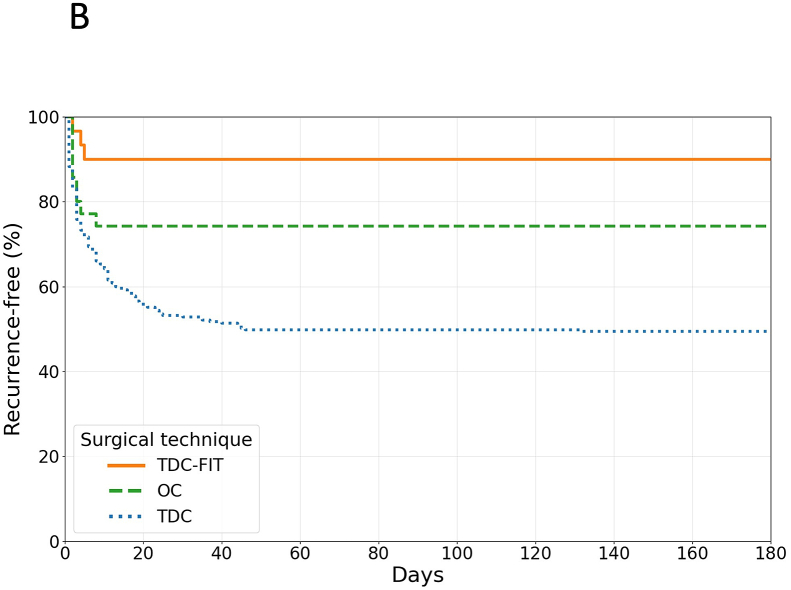
Kaplan-Meier curves illustrating recurrence-free survival following different surgical techniques for membranous cSDH. The y-axis indicates the proportion of patients without recurrence, and the x-axis shows time in days post-procedure (up to 180 days). TDC-FIT (orange) demonstrates superior recurrence-free survival compared to OC (green) and TDC (blue) (p < 0.001). Abbreviations: OC, open craniotomy; TDC, twist-drill craniostomy; TDC-FIT, twist-drill craniostomy with pressure-controlled fibrinolytic irrigation therapy.

### 3.5 Predicted risk model and actual recurrence rate

In internal validation within the TDC cohort, the baseline risk model showed moderate discrimination (AUC ≈ 0.72) and good calibration (calibration intercept ≈ 0 and calibration slope ≈ 1). When applied to the TDC-FIT cohort (n = 34), the model predicted a mean recurrence risk of 42.2% (SD 8.2) (median 45.5%; range 15.3–48.2) based on individual patient characteristics. However, the observed recurrence rate in this group was substantially lower at 8.8% (3/34).

In contrast, when the same model was applied to the TDC cohort (n = 672), the predicted mean recurrence risk of 30.2% (SD 19.0) (median 19.4%; range 13.2–89.2) closely aligned with the observed rate of 30.1%.

For the OC group (n = 39), the model predicted a mean recurrence risk of 43.1% (SD 8.3) (median 45.6%; range 14.7–48.0), while the observed rate was 25.6%.

### 3.6 Postoperative functional outcomes by surgical technique

**Fig. 2:**
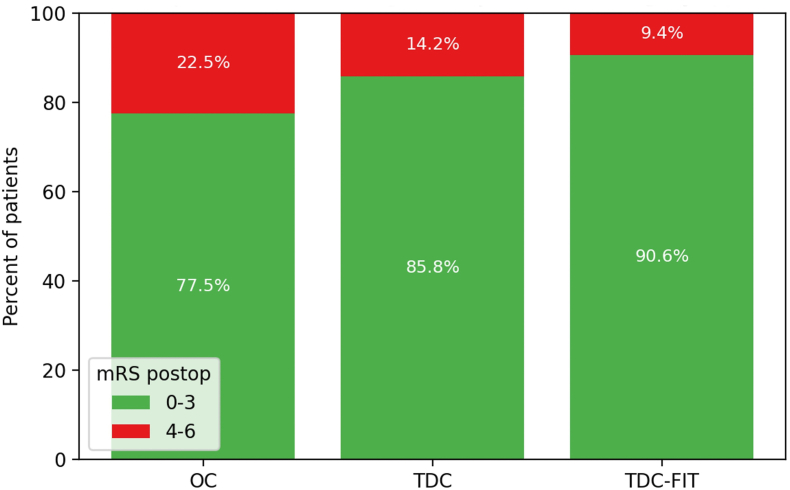
Distribution of postoperative mRS scores by surgical technique. The chart illustrates that there was a trend for higher rates of good outcome (mRS 0–3) in patients receiving TDC/TCD FIT compared to OC. Abbreviations: mRS, modified Rankin Scale; OC, open craniotomy; TDC, twist-drill craniostomy; TDC-FIT, twist-drill craniostomy with pressure-controlled fibrinolytic irrigation therapy.

To assess the impact of surgical technique on functional recovery, we analyzed postoperative mRS scores in the overall cohort and across the three principal surgical groups. In the total study population, postoperative mRS were generally favorable, with 84.6% with a mRS of 0–3. Among the three main surgical groups, the distribution of postoperative mRS scores revealed important differences. Patients treated with TDC (n = 698) and TDC-FIT (n = 34) had similar functional outcomes. Across the three surgical approaches, the proportion of patients achieving a favorable postoperative outcome (mRS 0–3) ranged from roughly 78 % in open procedures to about 91 % with TDC-FIT, yet these differences were not statistically significant (overall χ^2^ p = 0.25) (Fig. 2).

### 3.7 Adverse events

Perioperative adverse events (AE) occurred in 63 of 779 procedures (8.1%) and differed significantly by surgical technique (p = 0.019). AE rates were 7.4% for TDC (52/698), 8.8% for TDC-FIT (3/34), and 20.0% for OC (8/40). Pairwise comparison showed a significant difference only between TDC and OC (OR 0.32; p = 0.012), with no differences between TDC and TDC-FIT (p = 0.74) or TDC-FIT and OC (p = 0.21). CTCAE grade distribution did not differ across groups (χ^2^ = 4.64; p = 0.59), indicating similar AE severity (Table 2).

**Table 2:** Overall complication rates, severity distribution and AE by surgical technique. Data shows total procedures performed, absolute number and percentage of AE, and distribution of AEs according to CTCAE grading system (Grade 2: moderate; Grade 3: severe; Grade 4: life-threatening; Grade 5: death related to adverse event). Values represent the count and percentage of each AE type relative to the total number of complications observed for each surgical approach.

Surgical Technique	Total Procedures (n)	Adverse Events (n)	Complication Rate (%)	CTCAE Grade 2 (n)	CTCAE Grade 3 (n)	CTCAE Grade 4 (n)	CTCAE Grade 5 (n)
**TDC**	698	52	7.4%	13	4	18	17
**OC**	40	8	20.0%	1	0	5	2
**TDC-FIT**	34	3	8.8%	0	0	1	2

Surgical Technique	Complication Type	Count (n)	Percentage of Total Complications (%)
**TDC**	Drainage dislocation	13	25.0
**TDC**	Sepsis	13	25.0
**TDC**	Acute subdural hematoma	10	19.2
**TDC**	Subdural empyema	5	9.6
**TDC**	Intraparenchymal location	4	7.7
**TDC**	Meningitis	2	3.8
**TDC**	Myocard Infarction	2	3.8
**TDC**	Epilepsy	1	1.9
**TDC**	Covid Pneumonia	1	1.9
**TDC**	Stroke	1	1.9
**OC**	Acute subdural hematoma	4	50.0
**OC**	Subdural empyema	2	25.0
**OC**	Sepsis	1	12.5
**OC**	Others	1	12.5
**TDC-FIT**	Sepsis	2	66.7
**TDC-FIT**	ICP Problems	1	33.3

Abbreviations: AE, adverse events; CTCAE, common terminology criteria for adverse events; ICP, intracranial pressure; OC, open craniotomy; TDC, twist-drill craniostomy; TDC-FIT, twist-drill craniostomy with pressure-controlled fibrinolytic irrigation therapy.

### 3.8 Temporal trends

**Fig. 3:**
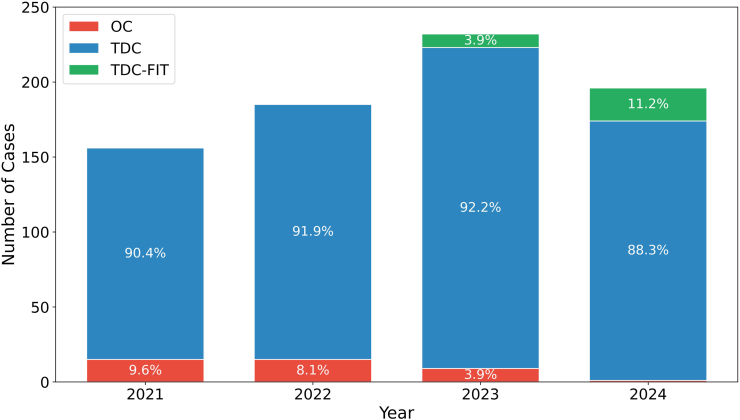
Stacked histogram showing the annual number of cases for each surgical technique: OC in green, TDC in blue, and TDC-FIT in orange. The y-axis indicates the number of cases, and the x-axis shows the year. Abbreviations: OC, open craniotomy; TDC, twist-drill craniostomy; TDC-FIT, twist-drill craniostomy with pressure-controlled fibrinolytic irrigation therapy.

**Fig. 4:**
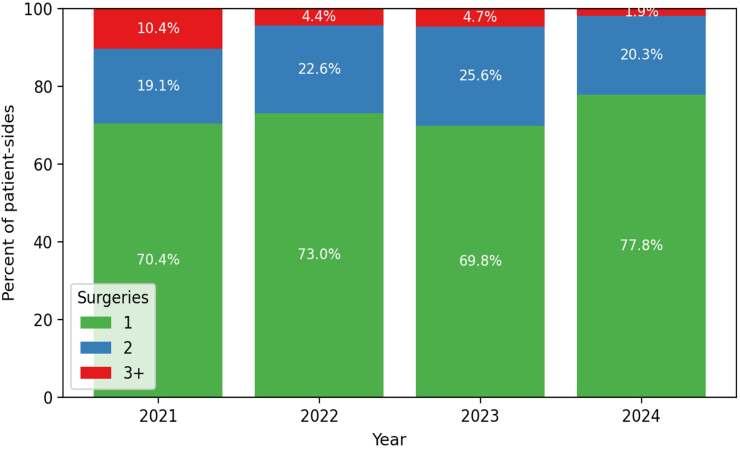
The image shows that cases requiring three or more operations fell steadily during the observed period, dropping from 10.4% in 2021 to just 1.9% in 2024. Throughout the same period, single-operation cases remained dominant, ranging from 70.4% in 2023 to 77.8% in 2024. Two-operation courses accounted for 19%–26% annually, peaking in 2023.

Over the study period from 2021 to 2024, there was a marked evolution in the surgical management of cSDH at our center. The temporal analysis of surgical techniques demonstrates a clear shift from OC toward minimally invasive approaches. In 2021 and 2022, TDC accounted for the vast majority of procedures, comprising 89.0% and 91.3% of cases, respectively, while OC represented 11.0% and 8.7%, respectively. TDC-FIT was not yet in use during these years. The introduction of TDC-FIT in 2023 was accompanied by a rapid uptake, with this technique representing 3.9% of cases in its first year and increasing to 12.6% in 2024. Concurrently, the use of OC declined steadily, falling to just 1.5% of all cases by 2024 (Fig. 3). Cases requiring three or more operations fell steadily during the observed period, dropping from 10.4% in 2021 to just 1.9% in 2024 (Fig. 4).

Between 2021 and 2024, roughly three-quarters of all patients received only a single procedure, while patients with two interventions accounted for about one-fifth. Cases requiring three or more operations fell steadily—from just over 10% in 2021 to below 2% by 2024 (χ^2^ p = 0.051) (Fig. 4).

## 4 Discussion

(version1) In this single-center cohort study spanning four years and comprising 779 surgical interventions for cSDH, recurrence emerged as a formidable and persistent clinical challenge, particularly in patients with membranous cSDH treated with TDC. Among the surgical modalities evaluated, TDC-FIT demonstrated superior efficacy, yielding the lowest recurrence rates both in the overall cohort and within the high-risk subgroup characterized by membrane-positive hematomas.

Conventional TDC with passive drainage continues to be favored for its minimally invasive profile, feasibility as a bedside procedure under local anesthesia, and tolerability in elderly or medically frail populations. Nevertheless, our findings corroborate longstanding concerns regarding its elevated recurrence rate, which in this series reached 30.0%, substantially exceeding that observed with BHC (Yagnik et al., 2021). OC, while associated with a somewhat reduced reoperation rate (25.0%), carries increased procedural complexity and perioperative morbidity, factors that have precipitated critical reappraisal of its role in contemporary practice. Notably, the marked decline in OC utilization at our institution following the introduction of TDC-FIT suggests a paradigm shift favoring less invasive interventions.

The clinical efficacy of TDC-FIT, combining the minimally invasive nature of TDC with adjunctive pressure-regulated irrigation and fibrinolytic therapy with urokinase, was underscored by the lowest recurrence rate of 8.8%. This benefit was particularly pronounced among membrane-associated hematomas, where TDC-FIT conferred a 4-5-fold reduction in recurrence compared to standard TDC.

When applied to the TDC-FIT cohort (n = 34), the model predicted a mean recurrence risk of 42.2% based on individual patient characteristics. However, the observed recurrence rate in this group was substantially lower, i.e. at 8.8%. In contrast, when the same model was applied to the TDC cohort, the predicted mean recurrence risk of 30.2% closely aligned with the observed rate of 30.1%, indicating good model calibration for this technique.

(Version 2) In this single-center cohort study of 779 surgical interventions for chronic subdural hematoma over four years, recurrence remained a significant clinical challenge, particularly among patients with membranous hematomas treated with conventional TDC. Among the approaches evaluated, TDC-FIT demonstrated superior efficacy, showing the lowest recurrence rates overall and in the high-risk subgroup with membrane-positive hematomas.

Conventional TDC with passive drainage remains attractive due to its minimally invasive nature, feasibility at the bedside under local anesthesia, and suitability for elderly or medically frail patients. However, its higher recurrence rate continues to be a limitation, whereas open craniotomy, although reducing recurrence modestly, carries increased procedural complexity and perioperative morbidity. The decline in open craniotomy use at our institution following the introduction of TDC-FIT reflects a shift toward less invasive interventions.

TDC-FIT, combining minimally invasive drainage with pressure-regulated irrigation and fibrinolytic therapy, yielded the lowest recurrence rates, particularly in membrane-associated hematomas. Predictive modeling suggested a substantially higher risk of recurrence for this cohort than was actually observed, highlighting the ability of TDC-FIT to mitigate recurrence independently of patient-specific risk factors. In contrast, recurrence predictions for conventional TDC closely matched observed outcomes, indicating good model calibration for standard techniques.

These findings indicate that while traditional risk factors accurately predict recurrence in conventional techniques, TDC-FIT appears to substantially reduce recurrence risk independent of patient-specific factors, making it particularly beneficial for patients with recurrent and/or membranous hematomas.

Emerging evidence has elucidated a range of clinical and pathophysiological factors associated with heightened recurrence risk following cSDH evacuation (Rauhala et al., 2020). Among these, the presence of organized membranes—potentially indicative of chronic inflammatory remodeling and neovascularization—has been robustly correlated with recurrence rates approaching 40–50%, particularly when treated with conventional TDC or BHC (Liu et al., 2019; Shimizu et al., 2020). TDC-FIT may uniquely mitigate these recurrence drivers through dual mechanisms: mechanical washout of hemorrhagic and inflammatory effusions and fibrinolytic degradation of fibrous septations and outer membrane scaffolds, which otherwise serve as substrates for reaccumulation (Bissolo et al., 2025a, 2025b; Doria-Medina et al., 2025). Importantly, pressure-regulated irrigation likely prevents sudden intracranial shifts that may precipitate membrane detachment or cortical collapse, a known risk factor for recurrence and neurological deterioration. This multifaceted mechanistic targeting may explain the dramatic recurrence reduction observed even in membrane-positive patients in our cohort.

Importantly, the adoption of TDC-FIT did not result in an increase in perioperative morbidity or AE. Furthermore, the capacity to safely perform subdural irrigation in cooperative patients on general wards obviates the need for ICU monitoring, thereby reducing healthcare expenditures and enhancing patient comfort. This approach is operationally less resource-dependent and logistically simpler relative to MMA embolization or BHC. Notably, TDC-FIT is performed as a single-step procedure, avoiding the need for additional interventions, procedural delays—now recognized as being associated with worse functional outcomes—or access to the endovascular compartment (Knuutinen et al., 2025). This eliminates potential complications associated with endovascular access, such as access site complications or adverse events related to embolization of arteries supplying vital cranial nerves or other critical structures.

MMA though generally well-tolerated and effective for the treatment of chronic subdural hematomas (cSDH), is not without disadvantages (Shafi et al., 2025). The procedure is resource-intensive, requiring specialized endovascular expertise, dedicated infrastructure, and appropriate scheduling capacity. The primary risks include access-related complications in an elderly patient population with vascular risk factors, potentially resulting in embolic events and cerebral ischemia. There is also a risk of treatment failure and early neurological deterioration necessitating additional intervention, particularly in patients with acute or subacute blood components within the hematoma, which has been identified as a strong predictor of embolization failure. Less frequent but noteworthy issues include unintended embolization of arteries supplying the retina or facial nerve causing transient amaurosis or facial nerve palsy. Additionally, MMA embolization may not address mass effect or rapid neurological decline, making it unsuitable as a sole therapy in certain acute situations. Overall, while complication rates are low (overall incidence under 4%), the procedure still carries small but significant risks that must be weighed against the benefits, especially in patients with specific anatomical variances or complex hematoma types.

While the overall cohort demonstrated good recovery, patients undergoing OC appeared to experience a modest but measurable increase in postoperative disability compared to those treated with TDC. There was a trend toward better outcomes with TDC compared to OC, suggesting a potentially clinically meaningful difference that merits further investigation.

**Fig. 5:**
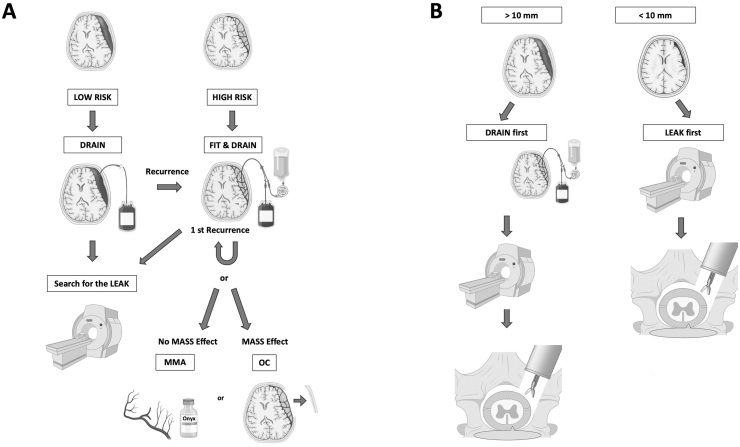
Flowchart of our proposed modern and minimal invasive decision-making algorithm for fast and cost effective chronic subdural hematoma treatment for better functional outcome. In case of SIH, patients will be trated with minimal invasive tubular approach. Abbreviations: cSDH, chronic subdural hematoma; MMA, middle meningeal artery; TDC, twist-drill craniostomy; TDC-FIT, twist-drill craniostomy with pressure-controlled fibrinolytic irrigation therapy.

Guided by minimally invasive principles and reduced logistical demands, we propose the treatment paradigm shown in Fig. 5. For patients without radiographic evidence of membrane formation and with a low anticipated risk of recurrence, TDC is recommended as the first-line intervention. Conversely, in patients with recurrent or clearly membranous hematomas—a group traditionally associated with higher rates of treatment failure—TDC-FIT may offer superior clinical outcomes and is therefore favored.

Whether the recurrence rate is comparable to that after MMA embolization should be investigated in future studies, however adjunctive MMA embolization is proposed as the next-line strategy in patients without mass effect, targeting the vascular contributors to hematoma persistence and recurrence. When hematomas are associated with mass effect, open craniotomy (OC) is indicated. Additionally, in patients with cSDH, evaluation for spinal CSF leaks must be mandatory. If a spinal CSF leak is diagnosed, targeted treatment of the leak should be undertaken (Overstijns et al., 2025). In accordance with recent studies management in the setting of cerebrospinal fluid (CSF) leakage should be tailored to mass effect (Knuutinen et al., 2025). This evolving algorithm departs from traditional paradigms that typically favored OC for complex or refractory cases. Instead, it emphasizes less invasive, approaches aimed at reducing recurrence while minimizing procedural morbidity.

### 4.1 Limitations

Several limitations warrant consideration. Given the partially retrospective design and the relatively small number of patients in the OC and TDC-FIT groups, potential confounding due to selection bias and operator variability cannot be fully excluded, although baseline characteristics were largely comparable among treatment cohorts. The limited duration of follow-up necessitates cautious interpretation regarding long-term efficacy and safety. Additionally, reliance on radiologic surrogates for membrane identification, while pragmatic, may not capture the full histopathologic heterogeneity inherent in cSDH membranes.

Given the retrospective design and the relatively small number of patients in the OC and TDC-FIT groups, these findings should be interpreted with caution. It is plausible that selection bias—such as the preferential use of OC in more complex or medically fragile patients—may have contributed to the observed disparities in functional outcomes.

Furthermore, we recognize that TDC is not widely established as the standard of care across neurosurgical institutions, primarily due to its well-documented association with elevated recurrence rates. In contrast, BHC remains the most commonly utilized surgical modality, supported by robust evidence demonstrating a reduced risk of hematoma recurrence when compared with TDC (Yagnik et al., 2021).

Despite these caveats, our findings substantiate the broader adoption of pressure-controlled fibrinolytic irrigation as a targeted, efficacious modality in cSDH management, particularly for patients with membrane or recurrent hematomas. As neurosurgical practice increasingly embraces precision-guided, minimally invasive interventions, TDC-FIT may represent a pivotal advance in attenuating the clinical and healthcare burden posed by this prevalent and challenging pathology.

## 5 Conclusion

TDC-FIT has demonstrated strong potential as a treatment strategy for recurrent cSDH, achieving substantially lower recurrence rates than conventional surgical approaches. At our center, it has become the preferred alternative to open craniotomy for managing recurrence, particularly in patients with membranous hematomas. In contrast to more resource-intensive techniques such as MMA embolization, TDC-FIT is less demanding in terms of infrastructure and scheduling, as it does not require general anesthesia and can therefore be performed with greater logistical flexibility. To fully validate its effectiveness and clarify its role within the evolving treatment landscape, future prospective trials and direct comparative studies with middle meningeal artery embolization are warranted.

## Funding

This research did not receive any specific grant from funding agencies in the public, commercial, or not-for-profit sectors.

## Declaration of competing interest

The authors declare the following financial interests/personal relationships which may be considered as potential competing interests:Marco Bissolo reports article publishing charges was provided by University of Freiburg Department of Neurosurgery. Marco Bissolo reports a relationship with University of Freiburg Department of Neurosurgery that includes: non-financial support. Marco Bissolo has patent pending to NO. If there are other authors, they declare that they have no known competing financial interests or personal relationships that could have appeared to influence the work reported in this paper.
